# The Role of Lithium-Ion Batteries in the Growing Trend of Electric Vehicles

**DOI:** 10.3390/ma16176063

**Published:** 2023-09-04

**Authors:** Alessandro M. Ralls, Kaitlin Leong, Jennifer Clayton, Phillip Fuelling, Cody Mercer, Vincent Navarro, Pradeep L. Menezes

**Affiliations:** Department of Mechanical Engineering, University of Nevada, Reno, NV 89557, USA; alessandroralls@nevada.unr.edu (A.M.R.); kaitlinleong@nevada.unr.edu (K.L.);

**Keywords:** Li-ion battery, electric vehicles, battery management system, battery, state of charge, solid-state battery

## Abstract

Within the automotive field, there has been an increasing amount of global attention toward the usability of combustion-independent electric vehicles (EVs). Once considered an overly ambitious and costly venture, the popularity and practicality of EVs have been gradually increasing due to the usage of Li-ion batteries (LIBs). Although the topic of LIBs has been extensively covered, there has not yet been a review that covers the current advancements of LIBs from economic, industrial, and technical perspectives. Specific overviews on aspects such as international policy changes, the implementation of cloud-based systems with deep learning capabilities, and advanced EV-based LIB electrode materials are discussed. Recommendations to address the current challenges in the EV-based LIB market are discussed. Furthermore, suggestions for short-term, medium-term, and long-term goals that the LIB-EV industry should follow are provided to ensure its success in the near future. Based on this literature review, it can be suggested that EV-based LIBs will continue to be a hot topic in the years to come and that there is still a large amount of room for their overall advancement.

## 1. Introduction

**Figure 1:**
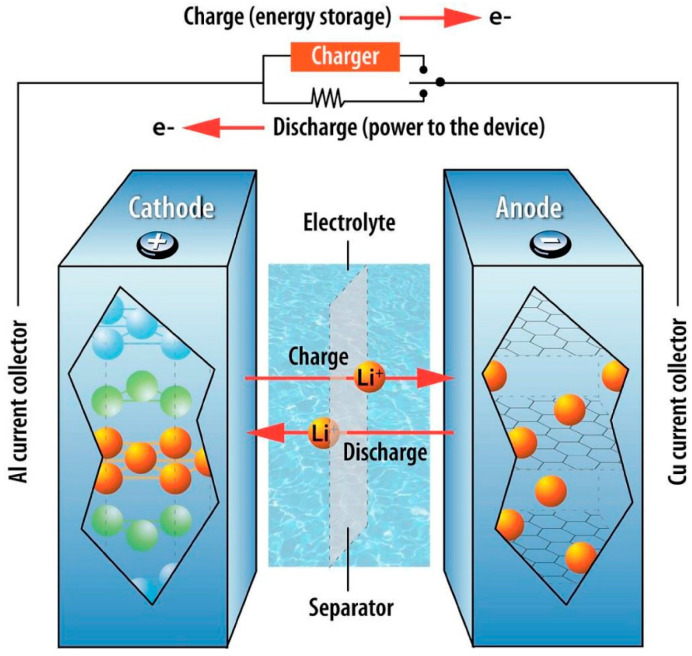
A depiction of the structural components for LIBs. Provided by Nzereogu et al. [6], Copyright 2022 under CC BY-NC-CD 4.0.

As electric vehicles (EVs) grow in popularity, the demand for lithium-ion batteries (LIBs) simultaneously grows. This is largely due to their impressive energy density-to-weight ratios (measuring at 120–220 Wh kg^−1^ [1,2,3]), which allows them to outperform other battery technologies such as lead–acid batteries (PbAB) and nickel metal hydride (NiMH) batteries [4,5]. Operating through a standard anode and cathode system, the ease of charge and discharge of electrons from Li^+^ ions allows for the generation of large amounts of energy, as shown in Figure 1. 

**Figure 2:**
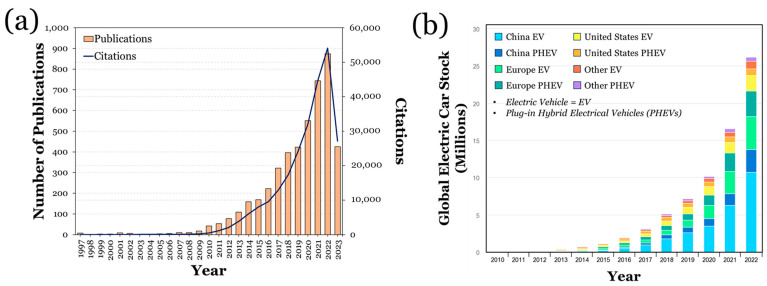
(**a**) A record of the number of citations and publications pertaining to LIBs from EVs from 1997 to July 2023, derived from the Web of Science [17]. (**b**) An illustration of the growing global stock of EVs and PHEVs from IEA [18]. This is a work derived by Ralls et al. from IEA material and Ralls et al. are solely liable and responsible for this derived work. The derived work is not endorsed by the IEA in any manner derived from.

Due to their structural advantage, LIBs have been shown to be the most widely used and reliable source of energy for electric vehicles (EVs) [6,7]. Evidence of this can be seen on an industrial scale, as a variety of automotive manufacturers (e.g., Tesla Motors) have largely utilized such batteries [8]. Within the academic community, extensive works pertaining to the study of LIBs in EVs have been noted. This can be quantitatively seen in Figure 2a, which enumerates the number of journal publications and citations pertaining to LIBs in EVs year to year. With data being provided by the Web of Science, the keywords “lithium-ion batteries” and “electric vehicles” were used for the initial search. All of the displayed data are listed from 1997 to July of 2023. It can be seen that around the 2010s, the interest in LIBs in EVs exponentially increased, which also coincidently relates to the global upsurge of consumer-owned LIB-based EVs [9,10,11,12]. To further demonstrate this trend, the EV market growth from 2010 to 2022 is shown in Figure 2b, where the stock growth of battery EVs and plug-in hybrid electric vehicles (PHEVs) across varying nations is shown [13]. Itis interesting to note that throughout the recent COVID-19 pandemic (which resulted in key competing companies such as Tesla laying off large amounts of employees [14,15]), the number of EVs sold continued to increase. A large amount of this reasoning can be due to the widespread introduction of EVs in the Chinese market, which resulted in overall greater sales [16].

However, despite their widespread usage, the safety concerns of potential LIB malfunctioning (due to the chemical reactions that occur at higher overheating temperatures) largely limit larger-scale industrial use [19,20]. Consequentially, there has been special attention to improving their resistance to degradative environments (e.g., mechanical-vibrational environments and/or submersed aquatic environments) [21,22,23]. Such concerns hamper the potential of LIBs, especially in EVs. In this work, the contemporary advancements within the EV-LIB field are detailed. Specifically, a novel and unique perspective of the general field of LIBs from economic, governmental, and manufacturing perspectives is discussed. Additionally, the technical role of LIBs in EVs is discussed alongside their current advancements and challenges. Suggestions on how to overcome current challenges in the field and to further grow the field are presented. Furthermore, timely suggestions for the short-term, medium-term, and long-term goals that the industry should follow to ensure future success are provided. Through these unique perspectives, the goal of this work is to give a holistic overview of the LIB industry to those actively in the industry or those barely joining.

## 2. Overview of EVs

To understand the importance of LIBs in EVs, the significance of EVs should be understood. Historically, EVs were initially introduced to fight the energy crisis in the early 1970s [24]. Acting as an eco-friendly alternative to oil-consuming automobiles, EVs acted as a way to achieve the same effectiveness as a combustion engine while solely relying on electricity [25]. At the time, due to their immense costs, there was a lack of attraction toward purchasing EVs. However, due to advancements in technology and the growing concern about climate change, there has been a gradual shift in the market for purchasing relatively more cost-effective EVs in the 2000s [26,27,28]. For instance, in early 2023, Audi announced that their entire automotive inventory will consist of EVs by the year 2029 [29]. Additionally, companies such as General Motors and Honda are also making commitments to similar goals [30].

Among the various countries that use EVs, the United States (US) has seen increasing growth due to the reduced prices of EVs and incentives offered by the national government. For example, in California, harsher emission regulations are being pushed to achieve a total of zero emissions. As of now, California has the goal to achieve 1.5 million EVs on the road by 2025, with each owner receiving a tax credit of $2500 [31]. Other US-based states like New York, Georgia, and Washington have been crediting numerous tax incentives. Washington, for instance, gives up to a 6.5% sales tax wave for purchasing EVs [32]. Outside of the US, China has also been showing strong motivation to push for alternative vehicles due to the increasing pollution problems in major populated areas [33,34]. In fact, as of 2021, a total of 3.2 million EVs were sold, encompassing more than half the number of cumulative EVs sold worldwide [35]. Japan also set up an ambitious goal (i.e., the Revitalization Strategy) to have EVs encompass 50–70% of the cumulative car sales in 2030 [36]. Various tax subsidies were also introduced in Japan, even after the historic Tōhoku earthquake in 2011 [37].

Despite these enticements that have allowed the EV market to grow at a substantial pace, there are various problems, which can be stemmed from their batteries [38]. For example, within current battery systems (e.g., nickel–cadmium (Ni-Cd), zinc/chlorine(Zn/Cl_2_), and nickel-zinc (Ni-Zn) batteries), they tend to have lackluster charging capabilities [39,40]. Additionally, they suffer from short battery life, thus creating an inconvenience for customers. Due to these issues, unwanted losses in energy, money, and inconvenience occur. To combat this, LIBs have been proposed as an alternative solution.

## 3. Lithium-Ion Batteries as an Alternative Battery Source

### 3.1. Historical Attraction toward Lithium-Ion Batteries in Electric Vehicles

**Figure 3:**
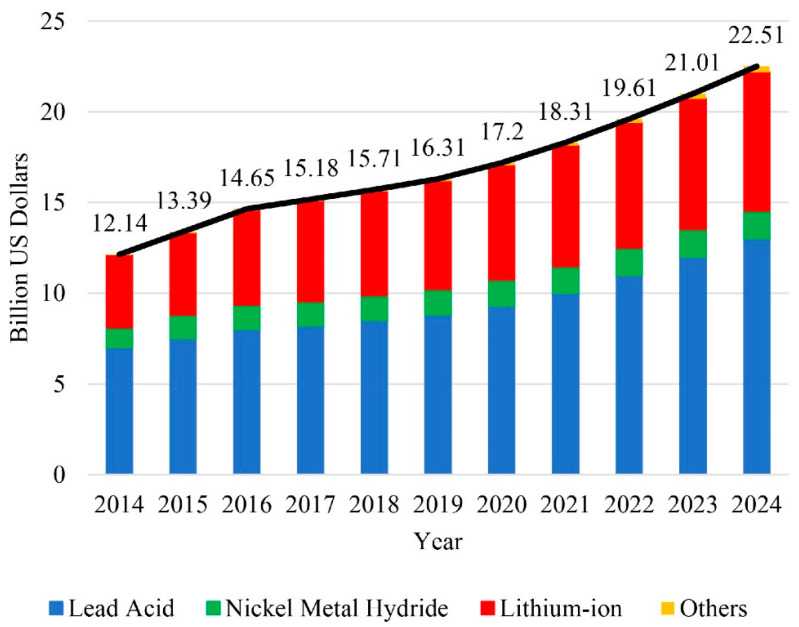
The market share of various EV-based batteries in the United States provided by Mohammadi and Saif [49]. Copyright 2023, under CC-BY-4.0.

During the aforementioned times of the 1970s, there was some experimentation on the feasibility of LIBs for EVs [41]. This trend began because of Nissan released their EV Altra model in 1997 [42]. However, in the beginning, it had lackluster sales due to its costly price ($50.99). In fact, only 139 units were ever sold [43,44]. However, afterward, a greater industrial interest began to take place to increase their operational efficiencies and decrease the total price cost, After some time of consistent development, large-scale automotive companies began to use LIBs with their vehicles [45]. Fast forward to current times, the market share of LIBs for EVs is gradually gaining more attention. For example, within the US market, LIBs have been becoming increasingly utilized due to the advancements in their capabilities (Figure 3). By 2030, it is also expected that 64% of the total light-vehicle sales will consist of LIB-based EVs will their combined amount encompassing 24% of owned light vehicles [46]. Even in China (which encompasses more than 50% of the current EV market [47]), it is predicted that nearly 37% of the vehicle market will also consist of LIB-based EVs [48].

### 3.2. Advantages of Lithium-Ion Batteries in Electric Vehicles

However, to further understand the attraction toward LIBs, it is important to understand their electrochemical characteristics. As previously mentioned, LIBs have an excellent energy density capacity. This is largely due to its cathodic-to-anodic ability to store high amounts of electricity (3842 mAh of electricity per 1 g of Li) [45]. Relative to other batteries (e.g., lead acid batteries, nickel–cadmium (Ni-Cd) batteries, and sodium–sulfur (Na-S) batteries), LIBs severely outperform them [50]. As a way to demonstrate this, the characteristic advantages of LIB batteries relative to other EV batteries (as well as the respective advantages and disadvantages of alternative batteries over LIBS) are shown in Table 1. As can be seen, despite any conceived advantages that other battery types have, they tend to suffer from their subpar performance. This is to be expected, as they are relatively mature technologies relative to LIBs.

### 3.3. Types of Commercialized Lithium-Ion Batteries in Electric Vehicles

To date, the existing types of LIBs in EVs can be subdivided into lithium iron phosphate (LFP), lithium nickel manganese cobalt oxide (NCM), and lithium nickel cobalt aluminum oxide (NCA) batteries [58,59]. These forms of EV-based LIBs differ mainly due to their elemental composition. With LFPs, the cathodic portion of the battery contains contents of phosphate, which tends to be safe material to use in the case of high-temperature conditions from potential thermal runaways [60]. From a structural perspective, LFPs are of olivine structure, which can resist unwanted lattice deformation and oxidation from the cyclic charge/discharge processes. Being relatively inexpensive and non-toxic to make, LFP batteries have wide usage in the EV industry [61]. In application, LFP batteries are predominately used in low-and-medium ranged EVs due to their longer lifetimes and environmental performance [62]. 

With EVs that operate in a medium-to-high range, NCM batteries are more desirable due to their impressive long-term electrochemical performance. This performance can be attributed to the material characteristics of manganese (Mn) and nickel (Ni). During application, the Mn creates spinal structures which can result in low internal resistance. Although Mn has low specific energy, the presence of Ni can improve specific energy, which allows for impressive energy and thermal characteristics [63]. Generally speaking, NCM batteries are also considered to be an eco-friendly battery option and see wide usage in the EV industry [64,65,66].

Aside from LFP and NCM batteries, NCA batteries are also used. With aluminum (Al) replacing Mn, the energy density of the battery is greatly increased. However, NCA batteries have a greater tendency for overheating and instability at elevated tempreatures, which present immense dangers for EVs [67,68]. Because of these downsides, NCMs are used less in today’s market and are gradually being phased out [62]. However, companies such as Tesla, Mercedes-Benz, BMW, Chevrolet, and Nissan still use NCA batteries, as they do not depend on Co, which has been reported to have cost fluctuations due to its uneven reserve distribution [68,69]. It can be speculated that in the future, as battery recycling becomes more mature, batteries of LFP and NCM origin will largely dominate the market.

Aside from Li-based batteries, attention toward Mg-ion batteries and Al-ion batteries for EVs is also taking place [70]. For Mg-ion-based batteries, the main attraction largely pertains to their overall abundance and inexpensiveness relative to Li-based batteries. Considering the environmental friendliness of Mg materials, it is likely that their industrial presence will increase with the concurrent global efforts toward having a circular economy. On the other hand, Al-ion batteries are also a point of industrial attraction due to their impressive energy and power densities [71]. However, given the amount of attention toward LIBs, their widespread application is limited. Despite this, the push toward eco-friendly batteries could potentially result in greater industrial usage of Al-ion-based batteries.

### 3.4. Impact of Current Policies and Regulations for Lithium-Ion Batteries in Electric Vehicles

Although statistical data can provide insight into the future development of the LIB-EV market, the direction of new policies and regulations from governments and international organizations also affects their future development. For example, in the European Union (EU), China, the USA, and Canada, automotive electrification with LIB technology plays a major role in their similar legislative goals toward carbon neutrality [72,73,74]. For the LIB-EV industry, the increase in government attention equates to the formation of funding opportunities and policies, which greatly contribute to their growth. For example, in the EU, the recently approved European Green Deal initiative will invest €1.8 trillion in funds to advance the technological advancement of EV-based LIBs. Specific focuses on research innovation, ensuring that material extraction practices are emitting less carbon, and proper storage are emphasized in these initiatives [75,76,77]. In the USA, the recent enactment of tax credits in April 2023 is also another method of incentivizing consumers to purchase EVs based on LIBs [78,79]. In Canada, performance incentives of up to $11 billion were recently enacted to fund the development of LIB manufacturing plants for EVs [80]. In Europe, the formation of lithium refineries from recent policies also contributes to the growth of the LIB-EV industry [81]. Of course, China has also been pushing similar policies, with a focus on investing in research and development to continue the advancement of LIBs in EVs, thus growing the market [82]. Based on these trends, it can be expected that the USA, Canada, the EU, and China will continue to be the frontrunners in helping expand the LIB-EV market in the years to come.

On the other hand, as the LIB-EV market continues to expand, growing concerns regarding the environmental repercussions of waste products are also taking place. For example, in the USA, the Environmental Protection Agency (EPA) has recently begun making efforts to promote the recyclability of and circular economy of LIBs in EVs [83,84]. On a governmental scale, funds from the Biden–Harris administration have also been released to accelerate LIB-EV recycling [85]. However, at present, there are no specific laws that require EV industries to recycle their LIBs [86]. As precious material reserves are also diminishing, the future of the LIB-EV market will face potential setbacks. We expect that the future of the LIB-EV industry will be geared toward investment in recycling technologies to sustain its growth.

## 4. Fabrication and Applications of Lithium-Ion Batteries in EVs

At this point, it is now evident that LIBs are the batteries of choice when it comes to EVs. In practice, there are various specific processes and roles that LIBs have in EVs. To understand these roles, details regarding the initial fabrication process to their performance as energy storage systems will be discussed in the following section. To collectively view the details of these processes, Table 2 will encapsulate the primary takeaways in this section.

### 4.1. Battery Fabrication

**Figure 4:**
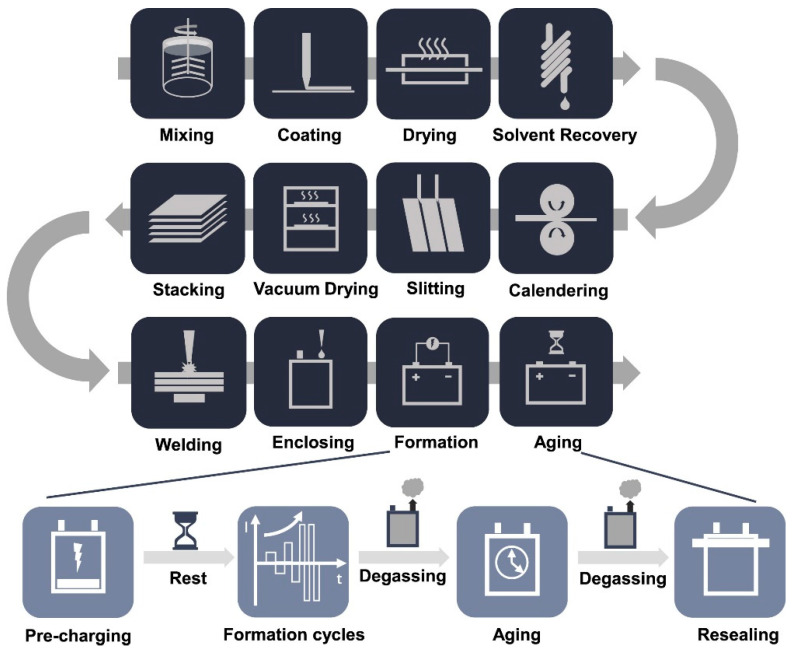
The typical manufacturing process of LIBs, provided by Liu et al. [99]. Copyright 2021 under CC BY-NC-ND 4.0.

Although LIBs in EVs have made exponential progress with their electrochemical performance, there is also a great deal of importance when it comes to LIB manufacturing, as it encompasses around 25% of the total cost of LIBs [87]. In current practices, LIB-EV manufacturing consists of a variety of complex and sequential processes, as shown in Figure 4. To begin, the selected material for the electrode alongside conductive additives, binders, and solvent are mixed. They are then applied as a wet coating to the current collector. The wet coating is then dried in a furnace to remove the solvent. Calendaring is then applied to ensure sufficient bonding strength between the current collector and electrode. The electrode is then slitted where they are vacuum dried to move unwanted moisture, which can result in hydrogen fluoride byproducts. Subsequent welding, enclosing, formation, and aging of the battery take place. The culmination of these processes has been reported to cost nearly $94 million/yr for an average LIB manufacturing facility using the BatPac model from Argonne National Laboratory [98]. 

**Figure 5:**
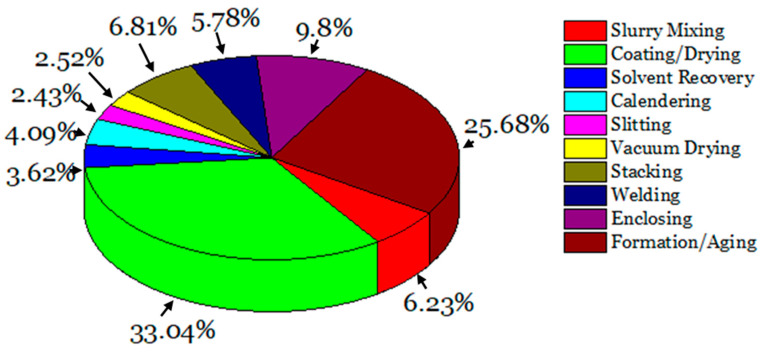
The percentage of cost for the various LIB manufacturing processes, with data derived by Paul et al. [98].

As of now, the most contemporary changes that are occurring in the LIB-EV manufacturing industry are reducing the costs of these processes. One easy way of doing so is to quantify the individual costs of each manufacturing process and to determine the processes that have the greatest operational costs. Using the manufacturing data from Paul et al. [98], the relative costs of each of LIB-EV manufacturing processes are shown in Figure 5. Among this list, the two highest cost processes are associated with coating/drying and formation/aging, generating ~$44.5 million in cost per year. In the drying process, a large portion of these costs are derived from the energy consumption of drying the organic solvent from the wet-coated electrode [99]. Drying is typically completed at high temperatures in an oven for prolonged periods. The temperatures will depend on the organic solvent that is used [100]. In the formation/aging process, the newly made battery will undergo low charging rates for up to three weeks to create the solid electrolyte interphase (SEI) from the Li ions, thus preventing the electrons from dissolving the electrolyte [99]. 

To reduce these costs, many new technologies are being explored. For example, to remove the need for solvents that require post-drying, solvent-free coatings through dry processing techniques have been proposed [101]. Dry coatings essentially apply electrode powders onto the surface by electrostatic-spray deposition (EPD). EPD can be thought of as a process that uses atomization by creating a voltage difference between the substrate and solution [102,103]. Aside from the ability to create electrodes two times the size of traditional electrodes [104], it is estimated that the drying speed can be improved by up to nearly 80% [105]. From a cost-savings perspective, that can reduce the costs from $13,984,000 to $2,796,800 based on the values from Paul et al. [98]. Regarding the formation/aging step, there are fewer alternatives at the current moment. If the charging rates are too high, a non-uniform SEI layer will form, decreasing the efficiency of the battery [106]. Methods such as applying a pulsed current charging have been proposed. However, such methods will vary based on battery chemistry and cannot be universally used [107]. It is suggested that novel coating techniques such as atomic layer deposition or plasma electrolytic oxidation be used to create a mimicked SEI layer, which can effectively reduce the wait times of aging from weeks to hours.

### 4.2. Battery Packing

Post-fabrication, the packing formation for LIBs is a critical step to ensure that they can safely operate and be transported (to EV assembly factories). Due to the highly volatile nature of Li, special packaging requirements have been established to ensure that damage due to a faulty battery is limited. The Electronic Code of Federal Regulations (e-CFR) lists federal requirements for the packaging and transportation of all Li cells within the United States (US). According to these regulations, Li cells must be completely encapsulated in inner packaging before being placed in strong, rigid outer packaging. These packages must then be capable of withstanding a drop test in any orientation without causing damage to the Li cells. Extra considerations must also be taken when transporting batteries by train or aircraft [88].

When LIBs are designed for use within EVs, extra care must be taken to ensure safety and reliability. EVs are intended to be used daily by the general public and will be present in highly populated areas. They are also at greater risk of sustaining damage due to the mechanical vibrations experienced by the car while driving and in the event of a crash could be ruptured if not properly packaged. Research into a more robust packaging system is ongoing, and current successful designs often focus on these factors [89,90]. Another growing concern is a thermal runaway, where LIBs can enter an uncontrollable heating cycle that is triggered by a high external temperature. For this reason, packaging designs should also include a thermal barrier to regulate the battery packs’ temperature [108].

**Figure 6:**
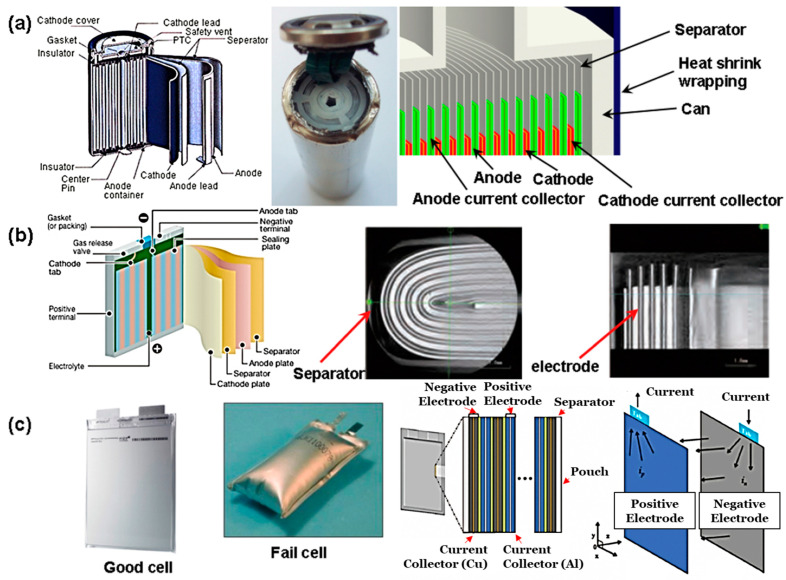
A depiction of the difference in (**a**) cylindrical celled, (**b**) prismatic celled, and (**c**) pouch celled LIBs, provided by Saw et al. [111]. Copyright 2016 with permission of Elsevier.

There are three main cell packaging types for LIBs in EVs. These packaging types consist of cylindrical, prismatic, and pouch cells, as shown in Figure 6. For cylindrical cells, the electrode is spiraled and placed within a cylindrical case, usually composed of aluminum, and placed sequentially within a modular pack. Although the cylindrical cell style can withstand deformation from vibrations and internal gas generation, the heat dissipation rate due to the surface area is a limiting factor [109]. Especially since EVs are designed for daily use, such an issue can be detrimental for long periods of driving. However, modern practices such as installing liquid intercooling systems can dissipate the excess thermal energy and improve LIB longevity. As an example, EV companies such as Tesla largely use cylindrical cells in their products [110]. 

Pouch cells are alternatively formed by having the electrodes stacked and placed in an aluminum-like pouch. Due to the geometry of the pouch cell and the ease of control for pouch thickness, the heat dissipation rates are much greater than in cylindrical cells [111]. However, as a result of tribological contacts due to cyclical rubbing motions during transportation/driving, unreversible plastic deformation can occur, which can rupture the pouch. 

For prismatic cells, the cathode, anode, and separator exist as long strips that are pressed and fitted inside a rigid container [91]. Packaging density is high in this type of cell, which makes this an excellent choice for EVs that require a substantial amount of battery power in a limited space. These battery cells utilize a pressure-activated disconnect and release vent to prevent overheating or over-pressurization of the cell. Prismatic cells can also be customized in design to a higher degree than other battery cells, which is useful when designing a battery for a specific vehicle. The casing for these cells requires metals with a heavy gauge to prevent bulging due to internal pressure buildup. In industrial environments, companies such as Mitsubishi use prismatic cells [111].

Among these three techniques, each one holds a series of advantages and disadvantages. From an operational perspective, the usage of cylindrical cells holds the most potential out of these three techniques. Part of this potential is due to their ability to withstand deformation, which is an advantage that other techniques do not have. Although the heat dissipation might be less, novel advances in battery management and liquid intercooling can easily mitigate these issues. However, they can be potentially difficult to design and implement in a manufacturing environment. It is suggested that the future directions of the field go toward using techniques such as immersion cooling. By using a fluid that has heat transfer rates, the latent heat of vaporization can enhance heat transfer rates, thus reducing the likelihood of overheating [112].

### 4.3. Energy Storage

**Figure 7:**
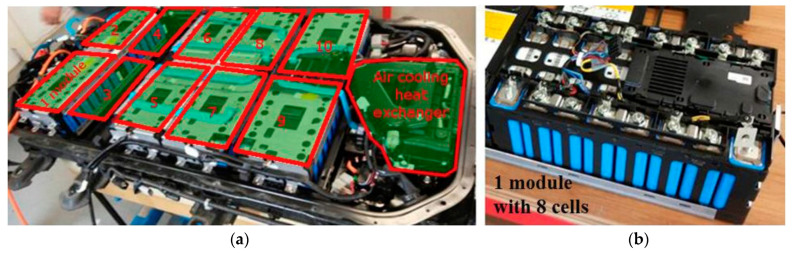
An assortment of (**a**) battery packs and (**b**) battery cells that are collected into a single module unit, provided by Cusenza et al. [113]. Copyright 2019, under CC BY 4.0.

After being packed together, the LIBs act together to conduct and preserve energy for EV application. They do so by being assembled into a module, which contains a variety of assorted battery cells, as shown in Figure 7. In application, as the temperature of the battery rises, so does the performance and capacity of the battery; this is caused by the chemical reactions that occur inside the battery. The reactions quicken thanks to the faster-acting molecules caused by the heat. This increases the performance of the battery, but it inversely shortens the life of the battery [92]. However, when these battery packs operate in colder temperatures, the same chemical reaction occurs much slower, reducing charging speeds and even reducing the maximum charge potential. It can be considered that cold temperatures limit the full potential of LIBs [93,94]. Many environments in the world where these EVs are being purchased can have colder temperatures, so careful considerations concerning their capacity and power loss over longer periods are to be kept in mind.

The average capacity of the battery cells inside of an EV today is anywhere from 35 to 63 Ah [97,114]. In the US alone, there are millions of EVs on the road today [46]. This puts extreme demand on the electric grid and the power storage facilities. The Department of Energy (DOE) also estimates that the annual costs for continuously charging a singular EV can span up to $2000 with a corresponding cost of $0.17 for one kW-h of energy [115,116]. Aside from the energy storage capabilities of LIBs, the ever-increasing rate of EV purchases will also require significant upgrades to the electrical grid. It can be insinuated that most of the money spent on upgrades will need to go to improving the battery capacity for quick charge and discharge capacitors.

### 4.4. Battery Management Systems

With the EV application of LIB storage systems, there are inherent disadvantages that limit their full usability. Specifically, LIB storage systems have been shown to have issues with durability, uniformity, safety, and excessive costs. To ensure operational success, LIBs must operate in a reliable and safe environment. However, external factors can alter this, which largely include unwanted temperature and voltage windows. These issues can lead to big safety problems and a decrease in battery performance [66]. Consequentially, a need for proper Li battery management is needed.

To understand how LIB storage systems can be controlled, it is important to understand the specific conditions and operating temperatures that they must adhere to. According to many, when the electrolyte reaches beyond 120 to 140 °C, the solid–electrolyte interface (SEI) begins to decompose, which can result in more exothermic reactions [117]. Specifically, the organic materials within the Li-ion battery will dissolve, which can result in combustible gases being produced. As can be insinuated, if the temperature increases further, there can be a violent reaction when the combustible gas mixes with the oxygen that is produced when the positive electrode decomposes. This can then cause fires or explosions [66].

**Figure 8:**
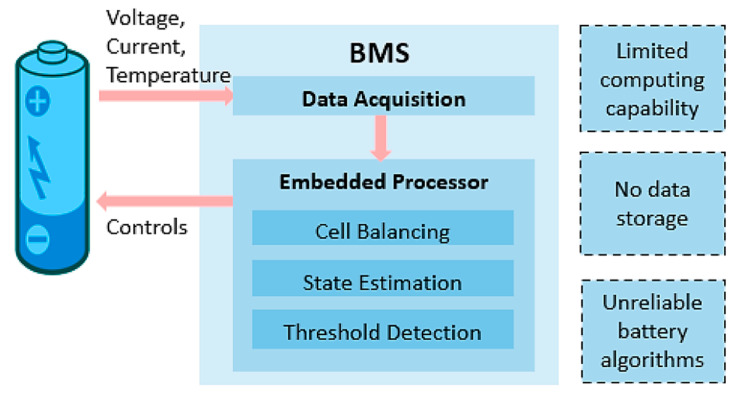
A visualization of the functional design of a BMS, provided by Tran et al. [95]. Copyright 2022 under CC BY 4.0.

These issues can now be solved with battery management systems (BMS). A BMS is a device that can be used to control the operational conditions of a battery. By doing so, the life of the battery can be extended with greater safety and the assistance to provide analytical information about the state of the battery. With this information, the energy consumption of the battery can be managed and reduced [118]. For reference, a visualization of a standard Li-based BMS system is shown in Figure 8, where the various operational features are highlighted. 

As previously discussed, as time passes, a battery’s ability to store energy will decrease. An indicator of this decrease is called the state of health, which is better known as SoH. The amount of time that is remaining for a battery before reaching its end of life is called its remaining useful life (RUL). A battery’s end of life is referred to as its EoL. A BMS is a protection circuit that also can help us to predict the battery’s SoH, RUL, EoL, available power, and capacity of a battery. All of this information helps to increase the safety and efficiency of the battery. For the application in EVs, a BMS that is based on models is more important, since EVs use much larger battery packs [4].

Upon closer inspection, a BMS collectively manages all energy storage and transfer in EV systems like charging and discharging, monitoring and balancing battery cell voltage, temperature control, battery protection, and fault assessment. The BMS uses the battery’s properties to control how the battery reacts to discharging and different changes in the environment [96]. The BMS also protects the battery from overcharging, undercharging, and also controls the temperature so the battery can operate safely. Having a BMS is especially important to a battery’s life because it can also find faults and assess faults in the battery to prevent any safety hazards in using the battery. In addition, LIB BMSs are designed to increase the lifetime, safety, and energy usage of LIBs. This system allows for the LIBs to estimate the state of charge (SOC) with low error [119]. 

#### 4.4.1. Smart Battery Management Systems

With new developments in EV-based BMS systems, it is possible to create a more complex system, which will increase the accuracy of the results. It can also lead to a higher load, may exceed the BMS limitations, and could also increase the costs [120]. However, there is currently other research being conducted about other designs for BMS that can improve the systems. One system proposed is a cloud-based smart BMS. The fundamental concept of cloud-based BMSs is to release the data storage constraints that standard BMSs have and to allow for the integration of improved battery algorithms [95]. 

Just recently, Yang et al. [121] introduced the Cyber Hierarchy Interactional Network (CHAIN) framework. This framework amasses EV/LIB BMS data into a cloud infrastructure, which can be used to determine the SOC and thermal conditions of the LIBs. Through a cloud-based infrastructure, the possibility of applying artificial intelligence (AI) and deep learning strategies can give a greater insight into future predictions of LIB life. The possibility of using deep learning methods for data mining can also improve operational aspects such as fault diagnosis, cell balancing, and state-of-estimations for various features of the LIB [121]. Of course, user-based features such as an API (application programming interface) and a UI (user interface) can allow for the driver to interact with potential warnings [95]. Based on these trends, it can be expected that deep learning strategies will continue to progress and be a hot topic within the field.

## 5. Materials Used in Li-Ion Batteries for EVs

Having a basic understanding of the roles of LIBs in EVs, it is also important to understand the implications of material selection in LIB efficiency and electrochemical performance. These materials mainly pertain to the anode and cathode electrodes used in the battery cell. In application, the cathode portion of an LIB is commonly composed of a material that can extract and re-insert Li-ions through the electrolytic solution in efficient quantities [122]. This is commonly known as intercalation and deintercalation. 

**Figure 9:**
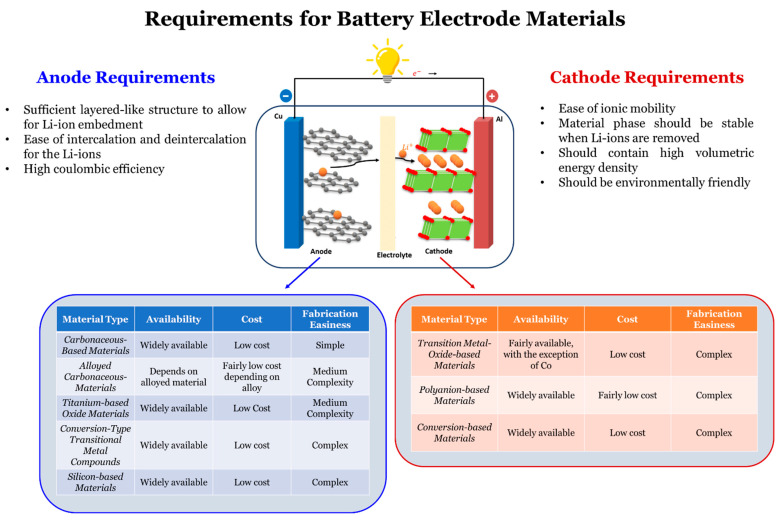
The various types of anode and cathode materials. alongside their requirements, availability, cost, and fabrication easiness, adapted from Spitthoff et al. [124]. All additional information was obtained from sources [124,125,126,127,128,129,130,131,132,133,134,135,136,137,138].

It is also important to ensure that the flux that is released from the cathode can be fully received by the anode materials. In cases where there is a flux difference, Li-ions are statically penetrated, thus blocking the charging and discharging of other Li-ions. This can increase internal resistance over time [123]. For this section, the key materials used for the anode and cathode components of LIBs alongside their contemporary advances are thoroughly discussed. As a reference, a comparative view of each anode and cathode material type, alongside their requirements, availability, cost, and fabrication easiness is shown in Figure 9. For additional reference, a summarization of these key sections is shown in Table 3. 

### 5.1. Anode Electrode Materials

As aforementioned, the function of anode materials is to allow for the discharge of lithium ions to the cathode, where they are effectively absorbed. As the battery undergoes various charging and discharging processes, the material and structural properties of the anode become increasingly critical to ensure long-term performance [165]. The anode material should have the capability of preserving the shape of the lithium-ions during charging/discharging, while also preventing the formation of a solid–electrolyte interface (SEI). The structural morphology is also important as it dictates the effectiveness of Li-ion intercalation and deintercalation [166]. In practice, the most common materials used for anode electrodes include carbon/alloyed carbonaceous materials, titanium-based-oxides, conversation-type transition-metal compound materials, and silicon-based materials. 

#### 5.1.1. Carbonaceous-Based Materials

Among the list of anode materials, carbonaceous-based (i.e., carbon-based) materials tend to be the most commonly used in the EV industry [167]. Among the existing list of carbon-based materials (e.g., single-wall and multi-walled carbon nanotubes [142,143]), graphite has seen the greatest usage due to its impressive intercalation and deintercalation capabilities [144]. These abilities stem from the two-dimensional (2D) structure that graphite has, as the Li-ions can easily infiltrate and store in between the layers [168]. Although graphene is widely used, graphene can suffer from low specific surface area due to the interactions of van der Waals forces during restacking [165]. To address this, many have induced defects and oxygeneated groups to form graphene oxide (GO) and reduced-graphene oxide (r-GO) [139,140,141]. By doing so, porous networks can be formed which can effectively promote greater Li-ion storage through greater accessibility. To date, the study of graphene nanocomposites is largely being undertaken to improve conductivity and total specific area [145]. 

##### 5.1.2. Alloyed Carbonaceous Materials

Although carbon-based materials such as graphite have impressive anode-performance capabilities, they tend to have somewhat lackluster long-term capacity retention and high susceptibility to thermal runaways [146]. The formation of SEIs enables these undesirable by-products, as the unused electrochemical energy transfers into heat, which gradually accumulates until combustion takes place [147]. To postpone these inevitable phenomena, alloying has been one proposed solution. Common alloy materials that have been reported include tin (Sn), silver (Ag), magnesium (Mg), aluminum (Al), and antimony (Sb). The advantage of using these materials is due to their increase in specific capacities and higher Li/Li^+^ onset voltages [6]. Among the existing literature, the most frequent way to form carbon-alloyed materials is through ball milling [169,170,171].

#### 5.1.3. Titanium-Based Oxide Materials

In recent years, the application of composite nanostructured titanium dioxide (TiO_2_) nanotubes has been one viable way of improving the electrochemical capacity and discharge rate of graphite anodes [148]. The attraction toward TiO_2_ lies in its high surface area and impressive electrochemical conductivity. When added as a composite, the insertion and disinsertion of Li-ions can be described as [149]:

(1) $$x{Li}^{+}+TiO_{2}+xe^{-}↔{Li}_{x}TiO_{2}$$

(2) $$y{Li}^{+}+C_{6}+ye^{-}↔{Li}_{y}C_{6}$$

**Figure 10:**
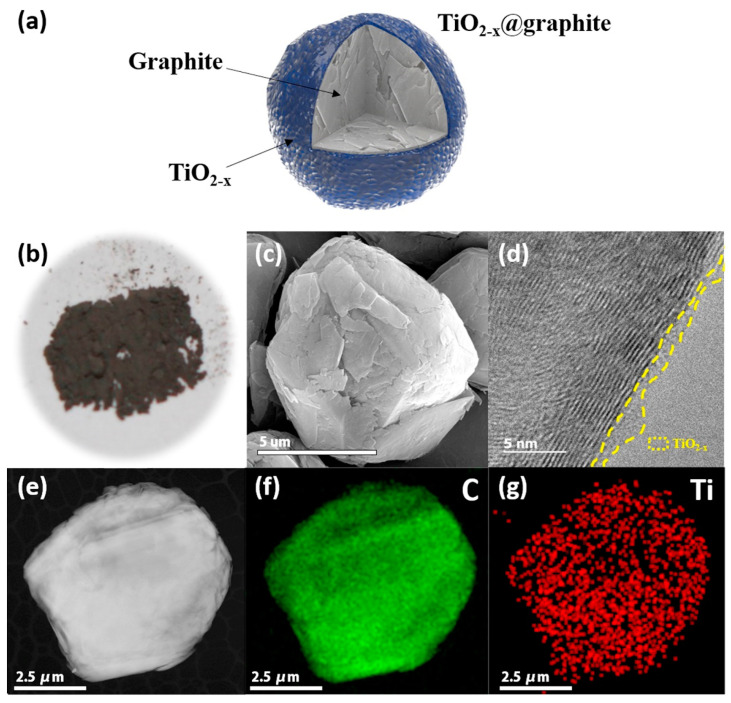
(**a**) Visualization of graphite being coated with TiO_2−x_; (**b**) macroscopic images of the graphite powder; (**c**) scanning electron microscope (SEM), (**d**) high-resolution transmission electron microscope (HR-TEM), and (**e**) TEM images of the coated graphite powder; energy-dispersive spectroscopy (EDS) maps of the (**f**) C and (**g**) Ti content along an individual graphite particle. Provided from Kim et al. [177], Copyright 2017, with permission of Elsevier.

As a visual example, a schematic illustration of graphite coated with TiO_2−x_ is shown in Figure 10. During cyclic ion exchange processes, the reversibility of TiO_2_ has been known to be quite impressive, as low volume expansion (within 3% to 4%) occurs [172]. When using TiO_2_, the polymorph that it also has dictated their electrochemical capabilities in LIBs. Investigations into the three primary polymorphs, anatase, rutile, and brookite, have been investigated as viable options for Li-ion insertion [173,174]. Among this list, the anatase phase has been one of the most widely used electroactive polymorphs. However, its complex structure inhibits optimal Li-ion insertion [175]. For the rutile polymorph, the undesirable cubic rocksalt-like structure that is formed during cyclical operation can reduce lithium diffusion by 10^−15^ cm^2^ s^−1^ [150]. In the case of the brookite polymorph, amorphization during cycling can result in similar irreversibility [176]. However, efforts to study the effects of other TiO_2_ phases such as the monoclinic bronze phase (TiO_2_(B)) have been increasingly studied as they have an open structural framework to allow for great Li-ion mobility during intercalation and de-intercalation [150]. 

#### 5.1.4. Conversion-Type Transition-Metal Compound Materials

**Figure 11:**
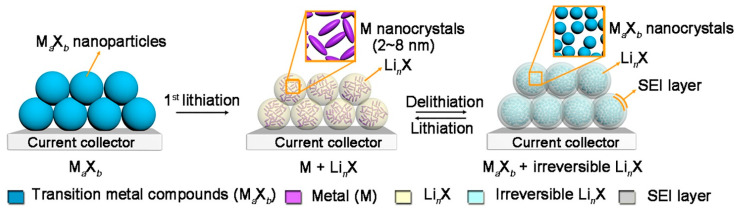
A depiction of the gradual growth of non-usable Li_n_X when M_a_X_b_ particles are used in anode LIBs. Provided by Lu et al. [151], Copyright 2018, with permission of Elsevier.

Another common anode material that is used is conversion-type transition metal compounds (CTAMs). Comprising materials such as transition-metal sulfides, phosphides, fluorides, nitrides, oxides, and selenides, the usage of CTAMs can act as a cost-effective and relatively straightforward alternative to LIB anodes. In addition, the lowered intercalation potentials do not suffer from dendritic growth, which can help improve the operational safety of EV LIBs [6]. To truly understand the viability of CTAMs, it is important to understand the implications of the redox reaction during LIB operation, which is most frequently referred to as the conversion reaction. This reaction is described as:

(3) $$M_{a}X_{b}+\left(b\cdot n\right){Li}^{+}+\left(b\cdot n\right)e^{-}↔aM+b{Li}_{n}X$$

where $M_{a}$ is the transition metal and $X_{b}$ is the compound. Based on this equation, its evident to see that there is thermodynamic feasibility of Li_n_X. However, the potential issue of the gradual accumulation of nonactive Li_n_X hampers long-term usability. This can be visually seen in Figure 11, in which the conversion reaction of CMATs to Li_n_X is shown. As an effort to mitigate this outcome, nanostructuring has been recently investigated to improve the reaction kinetics of CMATs [151].

#### 5.1.5. Silicon-Based Materials

**Figure 12:**
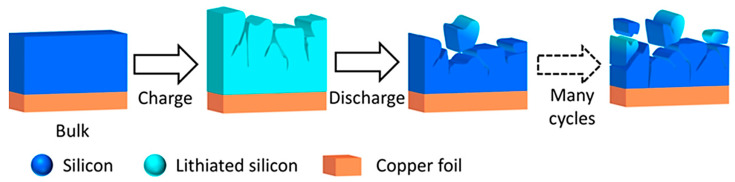
A visualization of the breakdown of the Si-anode material after cyclical discharging and re-charging LIB operation. Reprinted (adapted) with permission from Gonzalez et al. [155]. Copyright 2017 by American Chemical Society.

Lastly, Si-based materials have been one of the most recently studied alternatives for LIB EV anodes [152]. The attraction toward Si is due to its extremely high capacity, being reported to be nearly 10 times as large as traditional graphite [153]. However, the primary issue with Si is due to the volumetric expansion during cycling processes, thus resulting in a decrease in performance for the cell [154]. Furthermore, the gradual increase in intrinsic strain can result in cracking, which can eventually limit the long-term usability and eventual disconnection from the contacting current collector. A visualization of this process can be seen in Figure 12. Regardless, advancements in the forms of nanostructuring, pore structuring, binders, and composite additions have been proposed as viable strategies to mitigate such defects [155].

### 5.2. Cathode Electrode Materials

Aside from the anode, the cathode also has critical importance in the performance of LIBs. Similar to the anode, the cathode is based on intercalation materials. However, the primary use of cathodes is to host the Li-ions, which will be discharged during EV application. It is important to note that Li’s chemical potential should be lower in the cathode in contrast to the anode. This is due to the necessity of oxidation state stability while using a lower-lying energy band [156,178]. As such, the following subsections will detail the contemporary material advances in material selection for EV-LIB anodes.

#### 5.2.1. Transition Metal-Oxide-Based Materials

**Figure 13:**
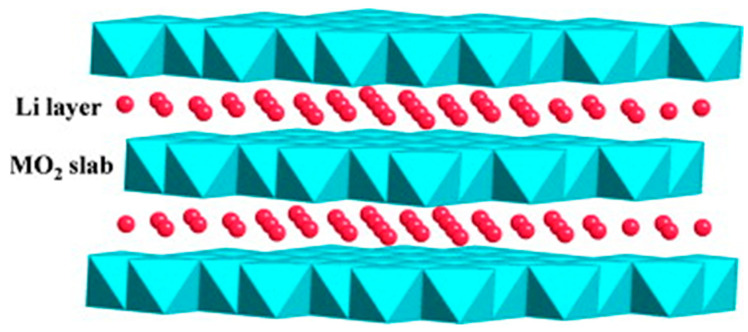
A depiction of the layered structure of LiMO_2_ cathode material containing Li ions. Provided by Xu et al. [156], Copyright 2012, with permission of Elsevier.

One of the most highly studied classes of cathode materials is layered oxides (LiMO_2_ where M can be Ni, Mn, and Co) due to their intercalation capabilities [157,158]. In addition, their ability to undergo oxidation when Li is transferred also makes them desirable [159]. The typical crystal structures tend to have a layered type of form, as shown in Figure 13. With these layers, a large number of Li-ions can effectively be stored between the 2D layers [156]. Notable metal-oxide-based materials that have been used within the literature consist of but are not limited to LiCoO_2_, LiNiO_2_, LiNi_0.8_Co_0.15_Al_0.05_O_2_, LiMnO_2_, LiNi_x_Co_y_Mn_z_O_2_, and spinal Li_2_Mn_2_O_4_ [164].

#### 5.2.2. Polyanion-Based Materials

Polyanion-based materials have also been used as cathode materials for LIB-based EVs. In contrast to transition metal-oxide-based materials, polyanions contain tetrahedral structural units (XO_4_)^n−^/(X_m_O_3m+1_)^n−^ (where X is typically S, P, Mo, W, or As) that are covalently bonded to MOx (where M is a transitional metal) polyhedral. The compounds of such polyanions largely consist of silicates, phosphates, fluorosulfates, fluorophosphates, and borates [137]. Essentially, the function of such polyanions is that they stay in certain positions of the cathode lattice, which can increase the overall cathodic redox potential [160]. However, issues in the form of low ionic conductivity exist due to distorted structural arrays. To combat such weaknesses, cationic doping and carbon coating have been proposed as viable solutions [161,162].

#### 5.2.3. Conversion-Based Materials

Lastly, conversion-based materials have been proposed as potential cathode candidates for LIB-based EVs. The uniqueness of conversion-based cathodes largely lies in their ability to break down and create chemical bonds throughout the cycling process. Most commonly, the conversion process takes place through one of the following equations:

(4) $$MX_{z}+yLi↔M+z{Li}_{\left(\frac{y}{z}\right)}X$$

(5) $$yLi+X↔{Li}_{y}X$$

Typically, M will consist of a transition metal ion (e.g., Mn^3+^, Cu^2+^, Co^2+^, Ni^2+^, Cu^2+^, and Fe^2+/3+^) and chalcogenide (e.g., Se^2−^ and S^2−^) or halogen (e.g., I^−^, Cl^−^, Br^−^, and F^−^) ions. The principle idea of reducing the metallic halides to a metal-like state will result in greater Li-ion conversion, which can attribute to theoretically high capacities [163]. As such, there is great interest in these types of materials for EV applications.

## 6. General Advancements and Challenges in Li-Ion Batteries for EVs

Considering the different uses and material selections for LIBs in EVs, there has been a great amount of evolution within the market since their initial introduction. Based on these current trends, the contemporary advancements and limitations of LIBs for EVs are thoroughly discussed. Specifically, the advancements in general LIB management, alongside the pursuit of the next generation of LIBs (i.e., solid-state li-ion batteries, lithium–sulfur batteries (Li-S-Bs), and Li-air batteries (Li-O_2_-Bs)) are thoroughly discussed. In addition, the current challenges in the field are also discussed. For reference, the key takeaways from this section can be shown in Table 4. 

### 6.1. Advancements for Li-Ion-Based EVs

#### 6.1.1. General Battery Management

As previously discussed, battery management is focused on the uncertainty of the internal chemical process that the battery is experiencing. This is routed in the fundamental idea that basic charge and discharge processes are affected by the environment [179]. To better predict these temperatures, mathematical models have been developed to understand the chemical reactions of the battery as well as the chemical degradation, thermal dynamics, and loss of active materials. Some examples of such mathematical models are that of John Newman’s usage of Stefan Maxwell equations to establish an explanation of the mass and energy transport of each species for each phase and component of the battery cell. By using these models, an understanding of the battery, when it is in a relaxed state compared to its state when heat is released, can be understood. It also helps them to understand the discharge curve of the battery. Other models that are used can infer data when the battery is in normal operating condition. It can model the power and energy contents of the system, which helps to analyze the lifespan of a particular battery cell. Although these systems are great in theory, it does not lead to an immediate conclusion of what the battery behavior is receiving. The complex environment in which the battery is operating makes it difficult to understand [180]. 

#### 6.1.2. Solid-State Li-Ion Batteries

**Figure 14:**
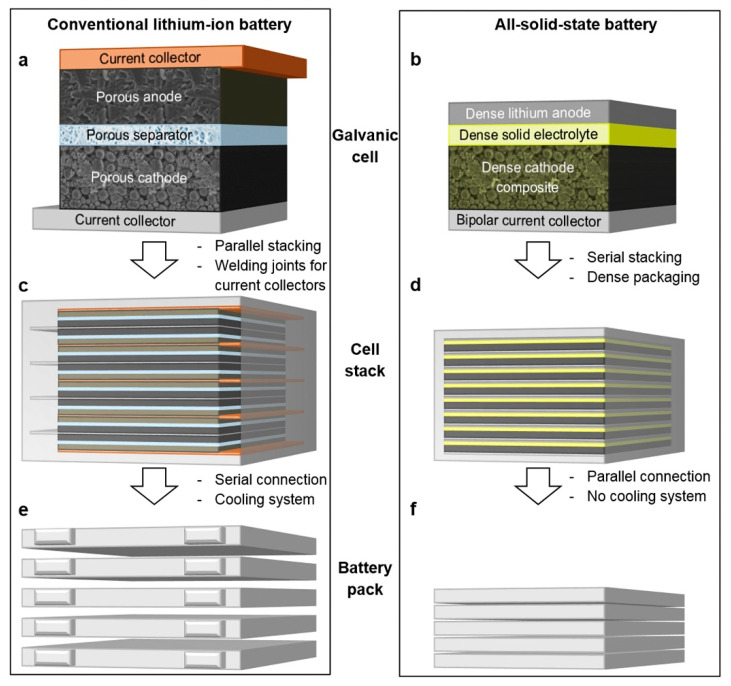
The structural difference between a state-of-the-art LIB and an SSLB at the (**a**,**b**) cell level, (**c**,**d**) stacked level, and (**e**,**f**) packed level, from Schnell et al. [182]. Copyright 2018, with permission from Elsevier.

One of the most exciting aspects of LIB technology is that there are numerous different varieties of lithium chemistries with different properties. One of the new and most exciting includes solid-state lithium-ion batteries (SSLBs) [181]. To transfer charge between an anode and cathode, an electrolyte must be used to transport the charge in the cell. Traditionally, liquid and organic electrolytes are used which require additional capacity and safety systems. Solid electrolytes address many of these downsides to provide improved performance and much safer operation by having a dense solid electrolyte that serves the simultaneous purpose of being an ionic conductor and electrical insulator [182]. This can be shown in Figure 14, which has a comparative overview of assembly for state-of-the-art LIBs and SSLBs. It can be seen that in a conventional LIB, the Li-cell contains porous anodic and cathodic electrodes as well as a porous separator (Figure 14a). A liquid electrolyte composed of conductive salts and aprotic organic solvents is used. If any of the solvents experience side reactions, aging and potential greater susceptibility to flammability can occur [203,204]. In the SSLB, the lithium anode sandwiches the solid electrolyte between a dense cathode composite (Figure 14b). When stacked, there is less space between the stacked cells with the SSLB (Figure 14d,f) relative to the traditional LIB (Figure 14c,e). Additionally, there is no cooling system for the SB due to the fewer number of organic components.

To further delve into the performance advantages of SSLB chemistries, having a solid electrolyte allows for batteries to be much safer from thermal runaway as liquid electrolytes are prone to leaking and catching fire. By using a solid electrolyte, unwanted leaks are effectively mitigated, thus resulting in greater safety from thermal runaways [202]. Solid electrolytes also happen to be much lighter than liquid, which means the electrolyte is much more space efficient. From this comes benefits such as higher specific energy, less weight for the car to accelerate, and simplified battery modules [183]. Another performance improvement is the lifespan of the battery. Liquid electrolytes will degrade over time while solid electrolytes are static throughout their lifespan [205]. 

#### 6.1.3. Lithium Sulfur Batteries

As it is already known, amongst all other rechargeable batteries, LIBs have been proven to have the highest energy density, thus being an integral part of large-scale energy storage for EVs [206]. However, over the past couple of decades, the cathodes in lithium-ion batteries have not been able to reach the required energy density necessary for EVs. Energy storage systems must have specific criteria that they should meet, which include low cost, long life, acceptable safety, and high energy [207]. With these values in mind, researchers have been investigating other cathode materials, such as sulfur, which can be used to increase energy density. Sulfur is one of the most abundant elements and has the highest theoretical capacity. Sulfur also has a low operating voltage and is more environmentally friendly than other materials [184].

**Figure 15:**
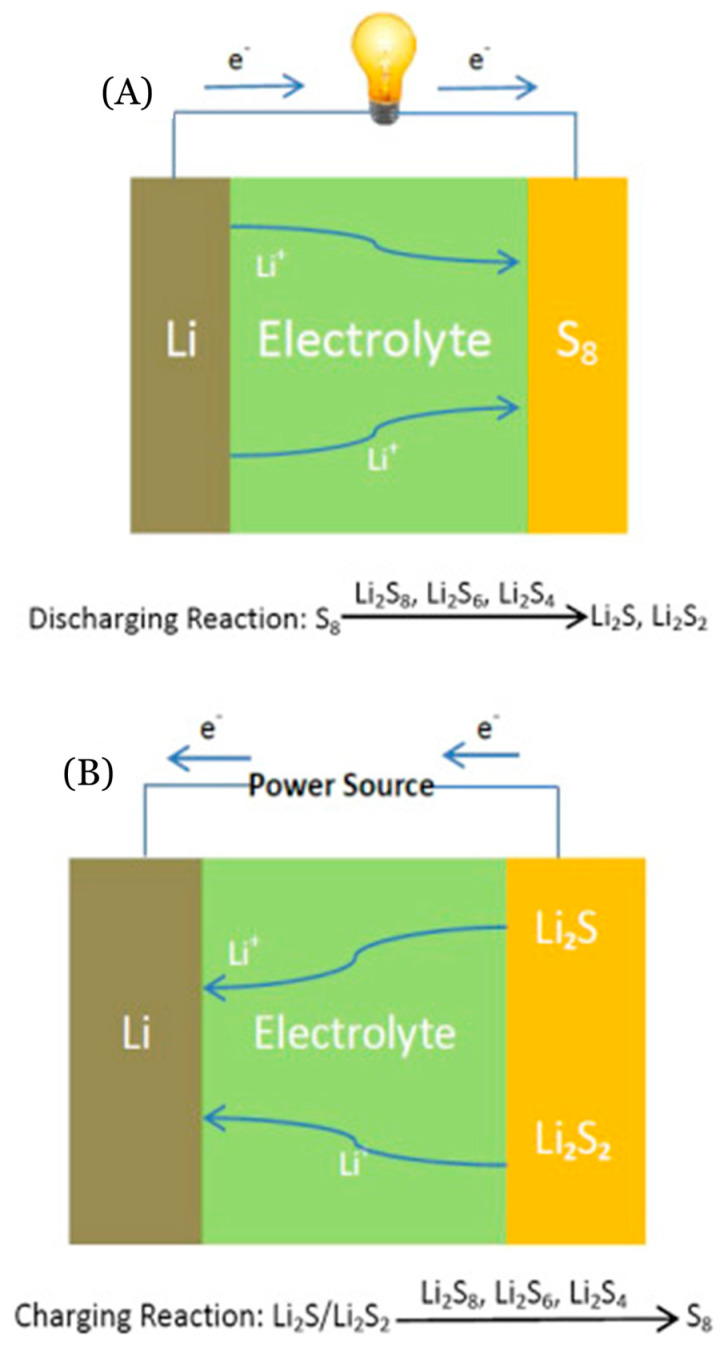
A schematic of the (**A**) discharging and (**B**) charging process for Li-S-Bs, from Chen and Shaw [186]. Copyright 2014, with permission from Elsevier.

A lithium–sulfur battery (Li-S-B) consists of four major components. These components include a Li metal anode, a separator, an electrolyte, and a sulfur-based cathode [184,185]. A schematic of a Li-S-B setup can be seen in Figure 15 for reference. During the discharge process, Li-ions are created from the oxidation of the Li metal which travels to the sulfur cathode through the electrolyte (Figure 15A). After traveling, Li forms Li-S-based compounds. For the recharge process (Figure 15B), the Li-ions go through a diffusion process which they return back to the anode [186].

Compared to traditional LIBs, Li-S-Bs have a multi-electron transfer chemistry and a higher theoretical specific capacity. It has also been determined by certain Li-S manufacturers that these batteries are going to have a practical gravimetric energy density that is more than twice that of LIBs. Sulfur also has a much lower cost, which provides the technology and EV industry with economic advantages. When designing Li-S-Bs, several design parameters need to be taken into account. Some of these parameters include the sulfur content, electrolyte type, electrode loading, and cycling conditions [184,207,208]. 

#### 6.1.4. Lithium–Air Batteries

In current times, lithium–air batteries (Li-O_2_-Bs) are a novel alternative battery that many are using over traditional LIBs. Although Li-O_2_-Bs are still being developed and researched in many ways, there are a variety of things about the battery that are well-known now. Li-O_2_-Bs are theorized to have the highest specific energy of any rechargeable battery coming at around 3500 Wh kg^−1^ [188]. For reference, this amount of storage is capable of driving an EV about 500 km. These batteries show promising results for energy storage and specific energy, although the overall understanding of the chemistry and electrochemistry of the battery is still growing [187].

**Figure 16:**
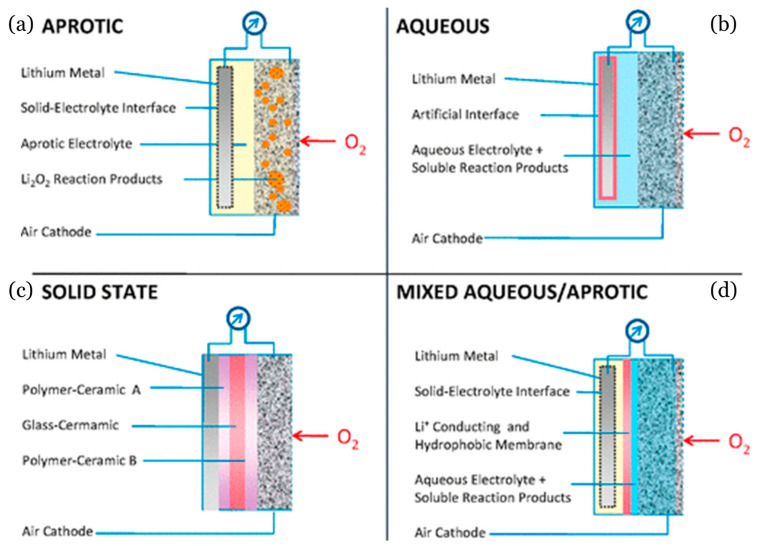
The various architectures of Li-O_2_-Bs, consisting of an (**a**) aprotic, (**b**) aqueous, (**c**) solid state, and (**d**) mixed aqueous/aprotic systems, provided by Girishkumar et al. [187]. Copyright 2010, with permission from ACS Publications.

Based on the structural difference of Li Li-O_2_-Bs, different safety concerns arise when used in EVs. Firstly, the Li material can form dendrites that can short-circuit the battery [209]. Secondly, if the dominant reaction of the product of the aprotic cells is mixed with an organic electrolyte, this could cause an issue in an accident, since the dominant reactor is a strong oxidizer [189]. However, previous works have been conducted and have concluded that there are no exothermic reactions that occur with LiO2 and common electrolytes at temperatures below Li-metals melting points. So in this case, for aqueous cells, the safety concern is negligent [187]. To date, there are four types of Li-O_2_-Bs [187]. Two versions are made of liquid electrolytes: a fully aprotic liquid electrolyte, and an aqueous electrolyte, and a mixed system with an aqueous electrolyte immersing in the cathode and an aprotic electrolyte immersing in the anode. The final type is similar to a solid-state battery with a solid electrolyte. For visualization, a schematic of the different Li-O_2_ chemical configurations is shown in Figure 16. It can be seen that all four are using a Li metal anode. To also achieve an ‘air-breathing’ capability, they also need a cathode to feed O2 without introducing contaminants. As for now, the only promising configuration has been the aprotic method. It has the best electrical rechargeability and has been the most focused on out of the four selections.

### 6.2. Challenges for Li-Ion-Batteries in EVs

#### 6.2.1. Development and Acquisition of Materials

As it is known up to this point, LIBs have their strengths when compared to other battery options: they can be charged and discharged reliably, have high energy densities, have high power-to-weight ratios, and perform very well in various environments. However, there are a number of challenges that LIBs face. First, LIBs are generally expensive to make, as extracting the raw materials needed to build them is extremely costly [190]. In a general LIB configuration, the most difficult and expensive material to extract is Cobalt (Co). Co is used in the cathode production of LIBs, and the price per kg has quickly increased from $30/kg to $90/kg in recent years, thus making it quite costly for EV applications [191]. 

Aside from the high cost of acquiring Co, there is also a need to improve the longevity of new materials selection. Specifically, materials that can decrease the overall surface area of LIBs while maintaining the same (or if not better) performance can greatly assist with their efficiencies as well as allow for them to fit into more confined spaces. Additionally, their recyclability, once they finish operation, should also be further explored. As of now, there is some attraction toward the field. However, the authors anticipate that this field will receive a much larger amount of attention in the upcoming years.

#### 6.2.2. Limited Lifespans

In addition to the current challenges, LIBs also have limited lifetimes. A major problem that has plagued the EV market has been consumers’ fear of battery life. LIBs use anodes, cathodes, and electrolytes to store energy from moving electrons [192]. Although they are the most reliable form of energy storage, they are not perfect. As of today, EV batteries typically last anywhere between 5 and 8 years depending on the amount of usage. Most lifespans in LIBs are limited because of the loss of Li-ions inside the walls of the electrolyte. Therese ions are often lost through side refractions that occur with the electrolyte to form compounds that trap Li, therefore leading to a reduction in Li-ions that can move between the electrodes [193]. Advancements are being made to stop the entrapment of these Li-ions in the electrolyte wall to increase the lifespan of these batteries. Nandan et al. [194] were able to add polymer composite binders to the silicon anode of LIBs, which improved the strength of these electrolyte walls and thus preventing ion stoppage. As previously mentioned, additional advancements in the selection of the material can offer more options when it comes to efficient and reliable LIB production/application. 

#### 6.2.3. Thermal Runaway

Another major and well-known concern with LIBs is their safety when it comes to unintended combustion/chain chemical reactions. In general, LIBs are extremely flammable. In some cases, the fire burning from the battery electrolytes can be more dangerous than petrol fires. The major problem is that batteries are full of flammable materials and chemicals, and the contained energy is capable of burning for long periods of time [22,69]. Due to this, car accidents where EVs catch flames can occur, which can be extremely dangerous [195]. Current advancements that are being made to strengthen the safety of LIBs include a battery cut-off function and lining battery packs with a thermo-protective layer to help prevent initial fire from an impact [196].

#### 6.2.4. Influence of Diffusion-Induced Stress and Capacity Fading

Another major concern for EV-based LIBs is the occurrence of capacity fading and diffusion-induced stress [210,211,212]. The issues are a by-product of how the SEI film is formed along the electrolyte-to-electrode interface. As aforementioned, the by-products of the electrochemical reactions result in the formation of this film. From one perspective, the SEI film can prevent the excessive consumption of the electrolyte due to the restriction of electron tunneling. However, despite this advantage, the SEI film will consume active lithium ions alongside the electrolyte material, which will result in capacity fading [210]. During capacity fading, the active electrode particles will become covered by the SEI film, which will result in greater ionic resistivity [211]. Mechanical degradation of the SEI film due to the interactions of electrode particles can also occur over time. Their interactions can result in less stability and fading of electrochemical performance. Efforts have been ongoing to fabricate durable SEI films and study the changes in stress. However, factors such as the structure/thickness of the SEI film alongside its composition and interactions with various particle sizes will vary its mechanical stability [212]. It is suggested that additional research be conducted in this field to further improve the operational efficiency of EV-based LIBs.

#### 6.2.5. Other Li-Ion Battery Issues

Aside from the previous points, there are also various downsides to newly introduced LIBs and their derivatives. For example, with SSLB technology, the primary downside is that it is still very new and needs more research to optimize the manufacturing process. The challenge with creating a solid-state battery is that the charge must be transferred conductively with the faces of the anode, cathode, and electrolyte material. This leads to unreliable electrical connections and pathways that can lower the efficiency and yield of the batteries. Some workarounds use a filler material between the materials to conform to the surface imperfections and provide better charge transfer. A small amount of liquid electrolyte can also be added to improve the transfer [202]. 

That being said, while many solid-state chemistries might have different features, each has shortcomings in a certain aspect of performance whether it be reliability, energy density, cost, and safety. With the current literature, some have reported that using a ceramic material to use as an electrolyte can allow for lower cost and lower environmental impact [198]. However, with more work on the material science behind the batteries, the various shortcomings of technology can be overcome. Eventually, solid-state battery chemistry could become suitable to fit all the future needs of the batteries in EVs.

On the flip side, when it comes to Li-S-Bs, although they are considered to be theoretically a better choice of battery, they also come with several challenges. Dating back to its first development, the initial goal of Li-S-Bs was to maximize the available energy through the complete utilization of sulfur. However, their formation of soluble polysulfides upon battery cycling results in a lowered Coulombic efficiency, which can result in degradation and potential issues [197,199,200]. As such, by improving the performance of solid electrolyte interphase (SEI) film, these detriments can be minimized. For example, in the work of Xiong et al. [201], it was found that adding a LiNO_3_ additive helped improve the detrimental shuttle mechanism in Li-S-Bs. Specifically, they assessed the electromagnetic behavior of these SEI films on Li-electrodes cycling in different solutions. Based on these findings, it was shown that the SEI film that was formed in the solution with Li-salts and electrolyte solution was relatively stable during cycling. These conclusions are made from a variety of experimental works, which allow the development of solutions to the known problems that exist with Li-S-Bs.

## 7. Recommendations for Overcoming Challenges for Li-Ion Batteries in EVs

To address these challenges, the following recommendations should be considered:

1. Efforts toward the synthesis of nanostructured composite materials should be made. By doing so, there can be less of a reliance on costly and limited materials such as Co.
2. Similarly, efforts toward recycling practices should be made. By doing so, limited and expensive materials can be re-used without the need for additional purchasing.
3. Application of external coatings such as plasma electrolytic oxidation (PEO) can reduce the likelihood of Li-ion entrapment, thus further limiting the formation of uneven and excessive SEI layers
4. Efforts toward cloud-based BMS and advanced cooling techniques should be investigated to prevent unexpected thermal runaways during driving.
5. Advancements in advanced, time-reducing, and cost-effective manufacturing techniques should be continued to increase the efficiency of LIBs for EV applications.

## 8. Conclusions and Future Directions

In this work, a unique and critical assessment of the growing advancements of LIBs in EVs was covered. Unique insights and predictions based on industrial, governmental, and academic trends were provided. Initially introduced in the 1990s, interest in LIBs has exponentially increased in industrial and governmental spheres within the past 5 to 10 years. This attraction is attributed to their impressive cathodic-to-anodic ability to store large amounts of electricity while emitting no carbon. As a consequence, advancements in the forms of battery manufacturing, battery management, and material selection have taken place. The novelties and most current trends presented in this review are as follows:

1. From an industrial perspective, three different trends are taking place. First, there is a trend toward manufacturing EV-based LIBs absent of Co. This trend is due to the cost fluctuation of Co due to uneven global reserves. Second, pushes toward reducing manufacturing costs/times of EV-based LIBs are taking place. The main areas of attention are toward the coating/drying and forming/aging processes. Lastly, trends toward advanced battery management systems, using features such as cloud infrastructures and deep learning algorithms, are rapidly occurring.
2. From a policy perspective, there is a global push toward the widespread usage of LIB-based EVs. However, concerns regarding the disposal of used LIBs are increasing. As a response, various countries such as the USA, Canada, and China are investing in advanced recycling technologies.
3. In recent years, there has been a great advancement toward novel materials for the anode and cathode electrodes. Although much attention has been on cathode electrodes, the general trends of using transition metals alongside polyanoion and conversion materials are taking place. Similarly, novel nanostructuring for LIB materials is also being investigated.

Although these novelties are currently taking place, discussions regarding the future strategies for widespread EV-based LIB usage, as well as collective short-term, medium-term, and long-term goals for the field should be made. The author’s suggestions for these points are as follows:

1. To allow for further widespread usage, it is suggested that advancements towards reducing the costs/time of LIB fabrication should be made. One option is to reduce the manufacturing processes needed for fabrication. Another option is to invest in low-cost and plentiful materials. By doing so, prices for EVs can be reduced, thus increasing their market share. These advancements can also prevent other technologies such as Al-ion/Mg-ion-based batteries from replacing LIBs for EV applications.
2. The short-term goal should be focused on reducing manufacturing costs/time. Being the most easiest and realistic short-term goal to achieve, focus should be made on the coating/driving and formation/aging processes.
3. The medium-term goal should be to achieve and commercialize efficient recycling technologies. By doing so, costly materials can be re-used, which can enable a circular economy.
4. The long-term goal should be to invest in more advanced material processing technologies. Specifically, attention toward nanostructuring and post-processing anode/cathode materials should be made.

By achieving these objectives, it can be expected that the LIB market will continue to keep the same pace of growth for the years to come.

## Figures and Tables

**Table 1:** A comparison of the advantages and disadvantages of LIBs compared to lead acid batteries, Ni-Cd batteries, and sodium–sulfur (Na-S) batteries.

Battery Type	Advantages	Disadvantages	References
Lithium-ion	Highest specific energy density (up to 220 Wh/kg) out of any existing battery Greatest cycle life out of any existing battery at 2000 cycles No periodic maintenance is required	Tend to have a relatively high cost Lack of general material availability, relative to other batteries Potential thermal runaways can occur due to overheating	[1,2,3,50,51]
Lead Acid	Relatively mature technology that has been time-tested More cost-effective Lower installation costs and higher recycling rates	Lackluster performance in harsher temperature environments Slow charging capability Low cycle-life	[52,53]
Nickel–Cadmium	Has a higher charge temperature range (from −20 °C to 65 °C Relatively good thermal stability	Well-known, so lessened research attention Varying degrees of efficiency A relatively costly process	[50,54]
Sodium–Sulfur	The utilization of nanostructures anodes can decrease weight and increase conductivity Lessened cost-to-performance ratio	Dendrite growth can occur, which results in low electronic conductivity Poor wettability along electrode-to-electrolyte interface, resulting in greater internal resistance	[55,56,57]

**Table 2:** The key takeaways from the role that LIBs have in EVs, from battery fabrication to battery packing, their energy storage, and the usage of battery management systems.

Lithium-Ion Batteries in EVs	Primary Takeaways	References
Battery Fabrication	LIB manufacturing encompasses around 25% of the total cost for LIBs Manufacturing process typically consists of creating a slurry mix, applying a coating, drying, slitting, vacuum drying, jelly roll fabrication, welding, formation, electrolyte filling, and lastly aging	[87]
Battery Packing	One of the most critical steps in having a successfully operational is having a safe packing formation Battery-packed LIBs must follow the requirements listed by *The Electronic Code of Federal Regulations* Three main cell packaging types consist of cylindrical, prismatic, and pouch cells	[88,89,90,91]
Energy Storage	As the temperature of the LIB increases, its performance also increases However, higher temperatures also reduce the capacity of the battery Colder temperatures also limit the number of redox reactions	[92,93,94]
Battery Management Systems	Battery management systems (BMSs) are used to control the operational conditions of the battery BMSs can also help predict the amount of life and efficiency a LIB has Current advancements for BMSs consist of adding cloud-based features and to further add application programming interfaces (APIs) and user interfaces (UIs)	[95,96,97]

**Table 3:** A summarization of the various advancements and challenges for LIBs in EVs.

Anode Electrode Materials		
Type of Material-Group	Primary Takeaways	References
Carbonaceous-based Materials	Graphite, single-wall, and multi-walled carbon nanotubes are largely used due to their impressive intercalation and deintercalation capabilities These materials are widely used in their 2D structures, being able to store large amounts of Li-ions To date, graphene nanocomposites and porous graphene oxide/reduced-graphene oxide materials are being investigated	[139,140,141,142,143,144,145]
Alloyed Carbonaceous Materials	Alloying elements such as tin (Sn), silver (Ag), magnesium (Mg), aluminum (Al), and antimony (Sb) are used to mitigate the formation of SEIs By adding these elements, the specific capacities and onset voltages of carbon-based anodes can be improved	[6,146,147]
Titanium-Based Oxide Materials	The addition of TiO_2_ is one viable option of improving the performance of anode electrodes This is largely due to their high surface area and impressive electrochemical conductivity Advancements toward studying monoclinic bronze phase TiO_2_ are currently being made	[148,149,150]
Conversion-Type Transitional-Metal Compound Materials	Conversion-type transition metal compounds (CTAMs) consisting of transition-metal sulfides, phosphides, fluorides, nitrides, oxides, and selenides are also used CTAMs have lowered intercalation potentials and do not suffer from dendritic growth	[6,151]
Silicon-Based Materials	Most novel anode material being studied Investigations toward Si are due to its high capacity, being 10 times greater than graphite The primary issue stems from volumetric expansion when repeatedly used Advancements in the forms of nanostructuring, pore structuring, binders, and composite additions have been proposed as viable strategies to mitigate such defects	[152,153,154,155]
**Cathode Electrode Materials**		
**Type of Material-Group**	**Primary Takeaways**	**References**
Transition Metal–Oxide-Based Materials	Transitional layered oxide materials have been increasingly studied due to their intercalation capabilities Typical structures tend to be in a layered-like form, which can effectively store large amounts of Li-ions prior to EV usage	[156,157,158,159]
Polyanion-Based Materials	The attraction toward polyanion-based materials is due to their tetrahedral structural units ((XO_4_)^n−^/(X_m_O_3m+1_)^n−^, where X is typically S, P, Mo, W, or As) which are covalently bonded to MOx (where M is transitional metal) polyhedral The function of these materials is to improve the cathodic redox potential due to their positioning within the cathodic lattice	[137,160,161,162]
Conversion-Based Materials	Conversion metals will result in transitional metal ions and chalcogenide or halogen ions undergoing a conversion-like process The conversion-like process consists of the breakdown of chemical bonds and the creation of new ones, allowing for long usability	[163,164]

**Table 4:** A summarization of the various advancements and challenges for LIBs in EVs.

Advancements in Lithium-Ion-Based Batteries for EVs		
Type of Advancement	Primary Takeaways	References
General Battery Management	Current attempts toward simulations can help better predict the electro-kinetics of LIBs By understanding these behaviors, the efficiency and performance of EV-based EVs can be improved	[179,180]
Solid-State Li-ion Batteries	Solid-state batteries reduce the need for liquid electrolytes for ion transfer Solid-state batteries can reduce the number of thermal runaways and potential leakage Solid-state batteries also have higher specific energy and longer lifespans	[181,182,183]
Lithium Sulfur Batteries	The utilization of sulfur can be a viable alternative to traditionally used LIBs Sulfur can greatly improve the energy density of LIBs Sulfur is also relatively cheap, thus reducing the price of LIB manufacturing	[184,185,186]
Lithium-Air Batteries	Lithium-air batteries (Li-O_2_-Bs) are one of the most novel LIBs in the current market Li-O_2_-Bs are theorized to have the highest specific energy Four types of Li-O_2_-Bs are used, consisting of (1) aprotic, aqueous, solid state, and mixed aqueous/aprotic	[187,188,189]
**Challenges in Lithium-ion-based Batteries for EVs**		
**Type of Challenge**	**Primary Takeaways**	**References**
Development and Acquisition of Materials	LIB-EVs are costly to manufacture due to costs from key materials such as Co There is a need to improve the material composition of LIBs to provide greater energy density and performance for EVs	[190,191]
Limited Lifespans	LIB-EVs tend to last up to 5 to 8 years, depending on their usage Side retractions result in Li-ion entrapment, which results in lesser Li-ion flow	[192,193,194]
Thermal Runaway	LIBs are extremely flammable Accidents where LIBs combust can be extremely dangerous Efforts to install battery cut-off functions and thermo-protective layers have been made to prevent thermal runaways	[22,118,195,196]
Other Li-ion Battery Issues	A challenge for LIBs is the lack of full comprehensive research on their abilities Formation of soluble polysulfides is one of the main downsides of LIBs Efforts to improve the solid–electrolyte interface (SEI) film for improved efficiency are being made	[197,198,199,200,201,202]

## Data Availability

Not applicable.
